# Supplementary material for: Analysis of PPARγ Signaling Activity in Psoriasis
Source: Int J Mol Sci. 2021 Aug 10;22(16):8603. doi: 10.3390/ijms22168603 (PMC8395241; doi:10.3390/ijms22168603)
Supplement: Supplementary file 1 [file ijms-22-08603-s001.zip › Supplemental materials_Analysis of PPARg signaling activity in psoriasis/Pathway models/Models images and html files/Differentiation of psoriatic T cells/189834.html]

macrophage activation


# Cell Process macrophage activation

|  |  |
| --- | --- |
| URN | urn:agi-gocellproc:0042116 |
| Total Entities | 1 |
| Connectivity | 1998 |
| Name | macrophage activation |

---

|  |  |
| --- | --- |
| ChildConcepts | microglial activation |

---

|  |  |
| --- | --- |
| Pathway | Cortisol in Resolving Inflammation |
|  | Th1-Cell Differentiation |
|  | Lipoxins and Resolvins in Inflammation Resolution |
|  | Th1-Cells Activation and Proliferation in Atherosclerosis |
|  | Atopic Dermatitis |
|  | T-Cells Differentiation Block in Psoriasis |
|  | Th1-Cell Function in Systemic Lupus Erythematosus |
|  | Immune Sustem Activation in Hashimoto's Thyroiditis |
|  | Graves Ophthalmopathy |
|  | Celiac Disease |
|  | Crohn's Disease |
|  | Paneth-Cell Function in Crohn's Disease |
|  | Th1-Cell Activation in Crohn's Disease |
|  | Macrophage Dysfunction |
|  | Dendritic Cell Dysfunction in Crohn's disease |
|  | Dendritic Cell Function in Ulcerative Colitis |
|  | Antigen-Presenting Cell Role in Asthma |
|  | Toll-like Receptors (TLR) in Periodontitis |
|  | Diabetes Induced Periodontitis |
|  | Estrogen Effects on Pregnancy Gingivitis |
|  | TNF Receptor Associated Periodic Syndrome |
|  | Mevalonate Kinase Deficiency |
|  | Blau Syndrome |
|  | Cryopyrin (NLPR3) Associated Periodic Syndromes |
|  | Familial Cold Autoinflammatory Syndrome 2 |
|  | Familial Mediterranean Fever |
|  | Alveolar Macrophage Negative Regulation Declined in COPD |
|  | Toll-like Receptors in Pyelonephritis |
|  | e |
|  | PPAR Psoriasis |
|  | Neighbors of macrophage activation |
|  | New Pathway (3) |
|  | 1\_Differentiation of psoriatic T cells |
|  | 1\_The role of Toll-like receptors in pyelonephritis |
|  | 1\_Insufficient activation of immune response in the middle ear epithelium cells in otitis media |
|  | 1\_Th2 cell response in asthma |
|  | 2\_Paneth cell dysfunction in Crohn's disease |
|  | 2\_Intestine epithelial cells drive immunological responses in ulcerative colitis |
|  | 1\_CD4+ T cell and epithelial cell response in celiac disease |
|  | 1\_Antigen-presenting cells promote islet dysfunction and immune system activation in T1D |
|  | Differentiation of psoriatic T cells |

---

|  |  |
| --- | --- |
| MedScan ID | 4142116 |

---

|  |  |
| --- | --- |
| Alias | macrophage activation |
|  | macrophage activations |
|  | activation of the macrophage |

---

|  |  |
| --- | --- |
| GO ID | 0042116 |

---
